# A multichannel feature-based approach for longitudinal lung CT registration in the presence of radiation induced lung damage

**DOI:** 10.1088/1361-6560/ac1b1d

**Published:** 2021-08-27

**Authors:** A Stavropoulou, A Szmul, E Chandy, C Veiga, D Landau, J R McClelland

**Affiliations:** 1 Centre for Medical Image Computing, Department of Medical Physics and Biomedical Engineering, University College London, United Kingdom; 2 University College Hospital London, United Kingdom

**Keywords:** radiation-induced lung damage, lung cancer, radiotherapy, computed tomography, longitudinal image registration

## Abstract

Quantifying parenchymal tissue changes in the lungs is imperative in furthering the study of radiation induced lung damage (RILD). Registering lung images from different time-points is a key step of this process. Traditional intensity-based registration approaches fail this task due to the considerable anatomical changes that occur between timepoints. This work proposes a novel method to successfully register longitudinal pre- and post-radiotherapy (RT) lung computed tomography (CT) scans that exhibit large changes due to RILD, by extracting consistent anatomical features from CT (lung boundaries, main airways, vessels) and using these features to optimise the registrations. Pre-RT and 12 month post-RT CT pairs from fifteen lung cancer patients were used for this study, all with varying degrees of RILD, ranging from mild parenchymal change to extensive consolidation and collapse. For each CT, signed distance transforms from segmentations of the lungs and main airways were generated, and the Frangi vesselness map was calculated. These were concatenated into multi-channel images and diffeomorphic multichannel registration was performed for each image pair using NiftyReg. Traditional intensity-based registrations were also performed for comparison purposes. For the evaluation, the pre- and post-registration landmark distance was calculated for all patients, using an average of 44 manually identified landmark pairs per patient. The mean (standard deviation) distance for all datasets decreased from 15.95 (8.09) mm pre-registration to 4.56 (5.70) mm post-registration, compared to 7.90 (8.97) mm for the intensity-based registrations. Qualitative improvements in image alignment were observed for all patient datasets. For four representative subjects, registrations were performed for three additional follow-up timepoints up to 48 months post-RT and similar accuracy was achieved. We have demonstrated that our novel multichannel registration method can successfully align longitudinal scans from RILD patients in the presence of large anatomical changes such as consolidation and atelectasis, outperforming the traditional registration approach both quantitatively and through thorough visual inspection.

## 1. Introduction

Non-small cell lung cancer (NSCLC) is one of the most common cancers in the UK. Historically, the prognosis for lung cancer patients has been poor, but advancements in treatments have caused mortality to decline (Howlader *et al* 2020). However, survivors of NSCLC can experience poor quality of life due to the toxicity of radiotherapy (RT) (Marks *et al* 2000, Fan *et al* 2001, Lopez Guerra *et al* 2012). The study of the negative long-term effects of radiation is becoming ever more important as patient survival rates increase. Radiation received during radiotherapy can lead to radiation induced lung damage (RILD). RILD is a time-dependent process, often split into two phases. Acute RILD or pneumonitis, is a phase of inflammation which occurs a few weeks or months after RT and is reversible. Chronic damage (pulmonary fibrosis) is the permanent scarring of the lung tissue that leads to impairment of oxygen transfer (Mehta 2005).

RILD can manifest as anatomical changes in the thoracic cavity such as consolidation, collapse and pleural effusion and can be detected in computed tomography (CT) images as changes in shape, density, texture and position between scans at different time points post-RT (Ikezoe *et al* 1988, Choi *et al* 2004, Veiga *et al* 2020). It is routine clinical practice for RT patients to be scanned every 3–6 months for up to 5 years after treatment. Studying the time-evolution of the radiological changes in these post-RT scans can provide a better understanding of RILD and potentially provide insight in the relationship between RT dose and clinical outcomes (Rosen *et al* 2001, Ma *et al* 2009, Simone 2017).

Existing scoring systems of RILD are limited, and there is yet no accepted methodology to quantify and measure RILD. Work has previously been done by our group to study global changes in the lungs (Veiga *et al* 2018, 2019). Objective image-based biomarkers were developed to quantify and evaluate such global changes as normal lung volume shrinkage, changes in lung shape, distortions of the diaphragm, etc. To calculate these biomarkers, it is only necessary to rigidly align the CT scans from different timepoints pre- and post-RT. Recently, we have been extending our suite of biomarkers to studylocal parenchymal tissue changes occurring due to RILD. However, to analyse the temporal evolution of the parenchymal changes it is necessary to align corresponding regions of the lungs. Due to the large anatomical distortions that can occur, accurate alignment cannot be achieved by simple rigid or affine registrations. Deformable image registration (DIR) can potentially be used to align the image from different time points, but the large anatomical distortions and considerable changes to the appearance of the images due to RILD make these registrations extremely challenging.

Traditional intensity-based DIR algorithms align the images based on the similarity of the intensity information in the images. However, the radiological appearance of healthy lung tissue is very different to that of damaged tissue and therefore it is unlikely that accurate alignment can be achieved using this intensity information. Furthermore, there can be large anatomical differences between the pre- and post-RT scans, such as tumour regression, tissue collapse, and fibrosis, which all violate the assumption of a one-to-one correspondence between the images made by most registration algorithms and make it difficult to obtain a meaningful alignment between the scans. Even when a one-to-one correspondence does exist, some manifestations of chronic RILD such as consolidation, atelectasis and cavitation can result in extreme geometrical deformations between the CTs. Consequently, attempting to align the pre-RT and the post-RT scans using traditional intensity-based algorithms usually gives unsatisfactory results. Veiga *et al* (2016) investigated and outlined many of the challenges in registering longitudinal RILD lung CTs. Additionally, the difficulties in working with clinical data from multiple institutions must also be considered. There can be large differences in image acquisition parameters and setup such as image resolution, field of view, patient setup and use of contrast that lead to differences in the scans that must be accounted for. Finally, even though follow up scans are usually acquired at breath-hold, baseline scans can sometimes be 4DCT scans, adding to the potential variability between time points.

Intensity-based DIR algorithms have previously been used to co-register pre- and post-RT scans. Cunliffe *et al* evaluated the accuracy of the Plastimatch (http://plastimatch.org) and the Fraunhofer MEVIS Fast deformable registration algorithms in registering CT scans before and 3 months after RT. They identified 8 out of 24 patients to have moderate to severe radiation induced changes and it was consistently shown that while the MEVIS algorithm performed better across most patients, the presence of RILD increased registration error for both algorithms (from 1.3 to 2.5 mm for MEVIS and from 2.4 to 4.6 mm for Plastimatch, almost 2 times the error in both cases). Spijkerman *et al* compared the accuracy of a Demons (Thirion 1998, Vercauteren *et al* 2009) and Morphons (Wrangsjö *et al* 2005) deformable registration algorithm with a rigid registration. Twenty-two NSCLC patient datasets were used, with pairs of pre and 3 month post-RT PET/CT scans. Two patient datasets were excluded due to the presence of atelectasis and pneumonitis in the follow-up scans. For the remaining datasets, while no major differences were observed between the Demons and Morphons algorithm, both showed improvement compared to the rigid registrations alone. Results were split into three groups according to alignment improvement with the major improvement group having a landmark error decrease from 9.5 ± 2.1 mm to 3.8 ± 1.2 mm, the minor improvement group from 5.6 ± 1.3 mm to 4.5 ± 1.1 mm and the insufficient improvement group from 13.6 ± 3.2 mm to 8.0 ± 2.2 mm. While there have been several studies attempting pre- to post-RT lung CT registration, registration of scans further than 3 months post-RT is still largely unexplored. This is likely due to the more dramatic changes seen at longer follow up scans as radiological changes continue to progress up to 24 months post treatment (Veiga *et al* 2020). Additionally, scans with significant consolidation and atelectasis are consistently excluded from these studies due to the known difficulties they pose to traditional intensity-based medical image registration algorithms.

More recently, the use of feature-based information along with intensity-based information has been explored as a mean of improving the performance of registration algorithms in aligning lung CTs by Guy *et al* (2018). They developed a DIR framework that employs a combination of the lung CTs, lobe segmentations, vesselness measure images and a mass preserving transformation to register longitudinal images in the presence of atelectasis with considerable success. Although scans with presence of atelectasis were purposefully included, the scan pairs were only a few weeks apart (pre- and mid-treatment). Lobe segmentation in moderately damaged lungs is in itself a very challenging task; in severely damaged or collapsed cases it is often impossible.

To address current challenges in the co-registration of CT scans in the presence of RILD, in this work we have developed a DIR method that aims to enhance and utilise salient features that are mostly unchanged between time points in order to successfully register scans that are 12 months apart. In our approach however, we discard intensity information to remove the impact that non-deformable parenchymal change would have during registration optimisation. This way, we aim to accurately align the consistent anatomical features between time points and hypothesise that the surrounding tissues will be driven into place accordingly. To the best of our knowledge, this is the first time that multichannel, feature-based DIR has been used to successfully align longitudinal lung CTs with extensive RILD.

## 2. Methods

### 2.1. Data

The patient data were derived from the IDEAL CRT trial cohort, a phase 1/2 nonrandomized multicentre trial that enrolled phase I and II NSCLC patients. 120 patients were enrolled to receive isotoxic tumour RT doses between 63 and 73 Gy in 30 daily fractions over 5 or 6 weeks, concurrent with 2 cycles of cisplatin and vinorelbine (Landau *et al* 2016). Fifteen patient datasets from this trial were selected for use in this study, with each patient having a CT scan at baseline (before RT was administered) and 3, 6, 12, and 24 months post-RT. These patients were selected so as to be representative of the changes seen over all patients. Most scans were acquired at breath-hold (deep inspiration) with the remaining being three-dimensional free-breathing CT or average 4DCT. There was both inter- and intra-patient variability in terms of scan acquisition parameters as well as scan resolution. The in-plane scan resolution ranged between 0.61 mm × 0.61 mm and 0.98 mm × 0.98 mm and the slice thickness from 0.5 to 5 mm.

**Figure 1.**
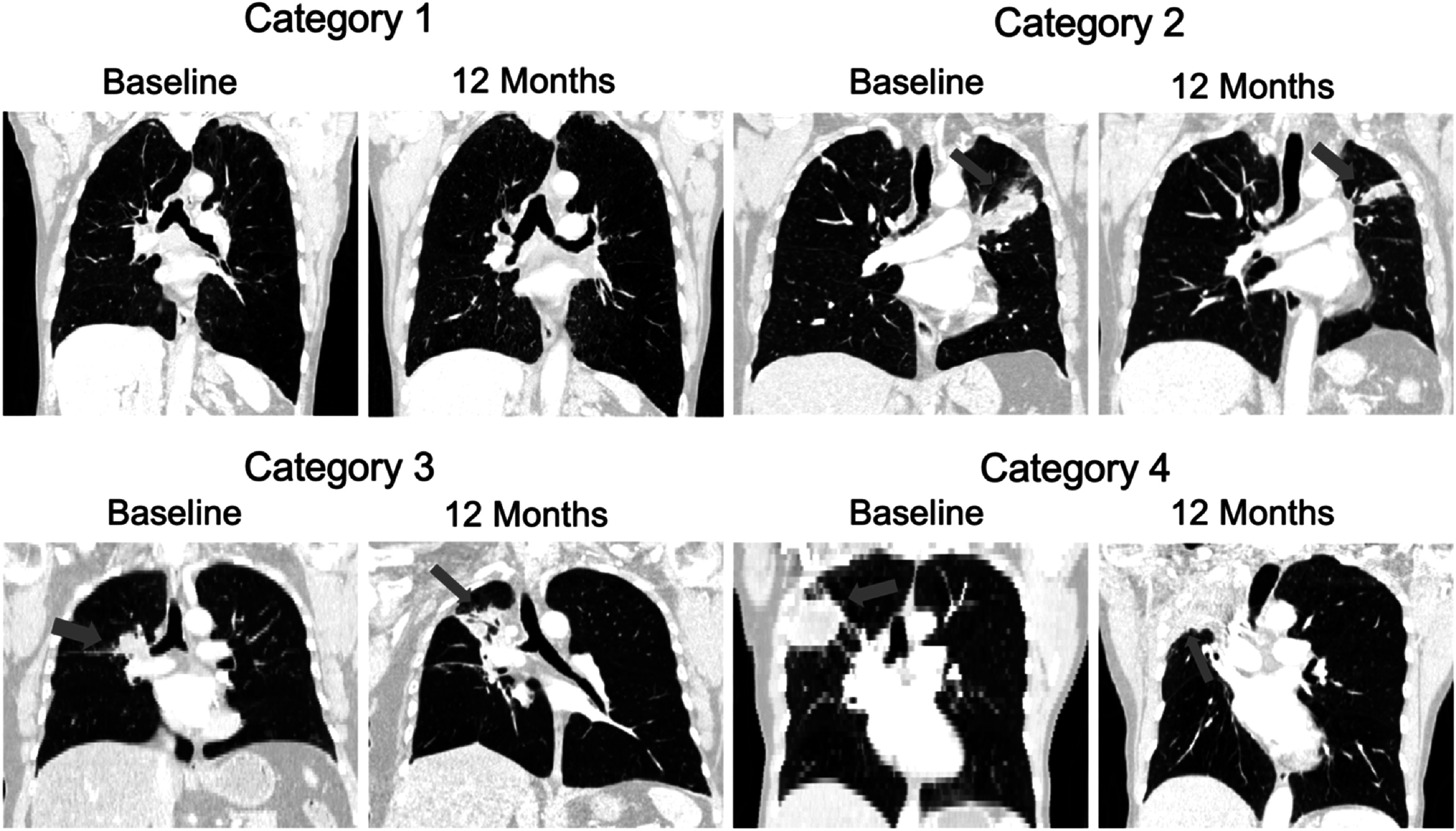
Baseline and 12 month follow up CT scan pairs of four patients with increasing registration difficulty. It can be seen that the scans in category 1 are very similar to each other, with most differences resulting from tumour regression in the left lung and inhalation level of the scans. In category 2, there are noticeable parenchymal and lung volume differences in the ipsilateral lung (left), but mainly contained in the upper lobe where the tumour is located. In category 3 such differences between the scans become more pronounced. Consolidated tissue is present in all three ipsilateral (right) lobes of the 12 month scan and the damage has extended past the region the tumour originally occupied. There are also changes in the size and shape of the ipsilateral lung between timepoints, with diaphragm tenting present in the follow up scan. In category 4, the most extreme changes are present with the upper right lobe completely collapsed in post-RT. The resolution is very different between the baseline (5 mm slice thickness) and follow up (1 mm slice thickness) scans, adding to the challenge in the registration.

The imaging data was visually reviewed, and each patient dataset was placed into one of four categories according to the expected difficulty of registration between the baseline and 12 month follow up scan. The categories corresponded to: (1) low, (2) medium, (3) high, or (4) very high estimated difficulty of registration. Four scans were placed in each of the low, medium and very high estimated difficulty categories and three scans in the high difficulty one. The criteria considered for this scoring were based on the severity of radiological changes present and scan quality, including: the extent and location of consolidation present in the follow up; the presence and extent of pleural changes and/or atelectasis in the follow up; the differences in lung shape and size; the presence of other anatomical changes associated with fibrosis such as tenting of the diaphragm and mediastinal shift; the presence and extent of cavitation in the follow up; the presence and extent of motion artifacts; whether an image was stitched from multiple scans; the difference in resolution between scans; and how difficult it was to visually determine correspondence between scans. An example from each category is presented in figure 1.

### 2.2. Feature representation and pre-processing

The principal idea behind our approach is to enhance and utilise features that are salient and mostly unchanged between time points while ignoring any information where there may be no clear one-to-one matching that could wrongfully guide the registration. The selected salient features include the lung boundaries, the airways, and the vessel tree.

#### 2.2.1. Lung boundaries and main airways

Segmentation of the lungs and the airways were performed semi-automatically as described in Veiga *et al* (2018), by using the open-source Pulmonary Toolkit (PTK) (Doel 2012) to generate automated results. PTK first performs Gaussian smoothing and thresholding on the CT images. Airway segmentation starts with region growing from a point in the trachea and a 26-way connected component approach is applied to organise the airways into a tree-like structure with multiple generations. The segmented airways are removed from the thresholded initial lung masks. The next step separates the lungs into left and right lung, which is initially approached with reversable opening of the lung masks. If that approach is unsuccessful, the watershed method is applied. The final step smooths the separated lungs individually using morphological operations. All segmentations were subsequently manually edited by a radiation oncologist or a medical physicist (EC/CV) using ITK-SNAP (Yushkevich *et al* 2006).

**Figure 2.**
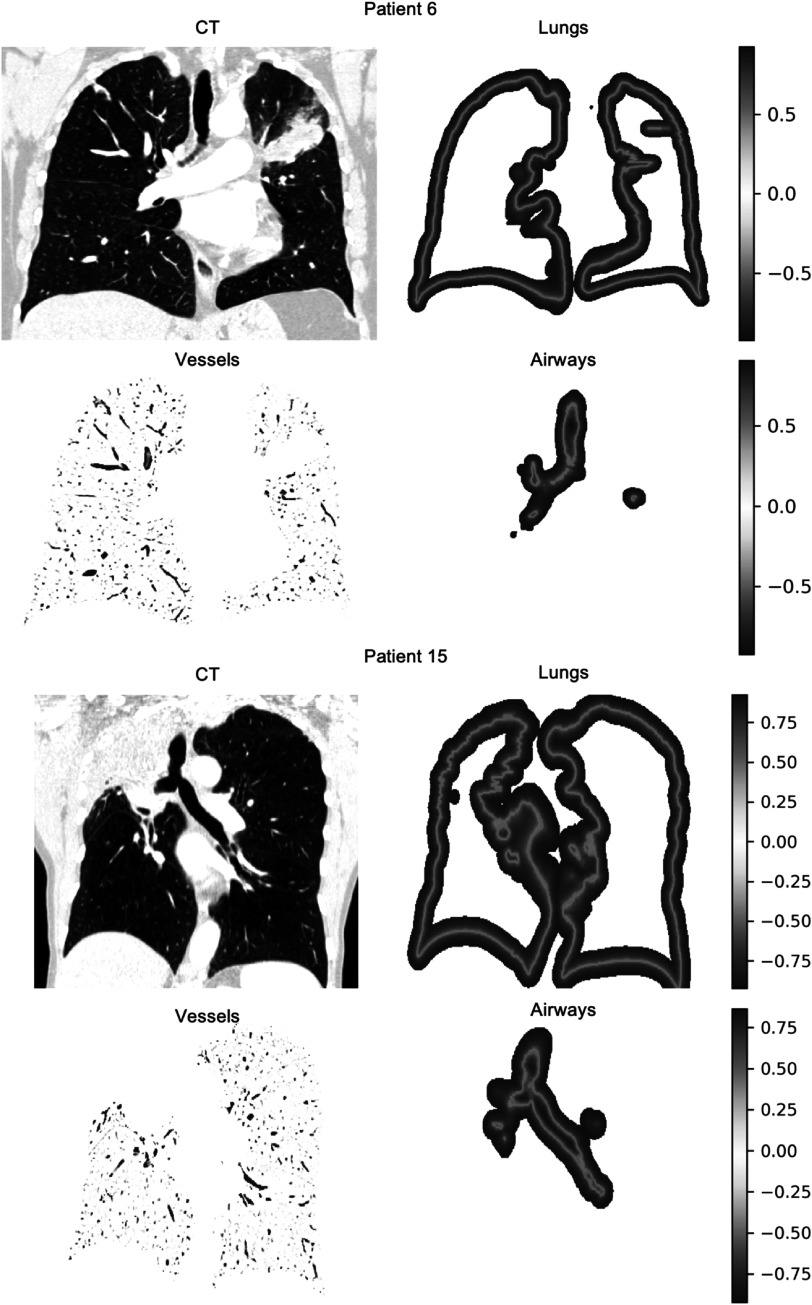
Feature images of patient 6 at baseline (category 2) and patient 15 at 12 months (category 4). From each CT the lung distance transform, airway distance transform and vesselness are extracted. Note that atelectatic tissue (patient 15, upper right lobe) is encompassed in the lung feature image.

Instead of using the binary segmentations directly, we opted to use signed distance transforms derived from the segmentations. Intensity based registrations are driven by the intensity gradient in the images being registered, but for binary segmentations the image gradient is 0 everywhere except directly at the boundary of the segmentations. This can cause problems in cases where the structure is significantly misaligned to start with, as no gradient information is available in the segmentations to drive the structures together. A common approach to combat this, is to smooth the binary images before registration, but this also has the effect of smoothing the segmentation boundary. Therefore, by using signed distance transforms instead, we introduce the necessary gradient while simultaneously enhancing the structure boundary. However, using a Euclidean distance map would cause the registration to align all voxels that are equidistant from the segmentation boundary, regardless of how far they are from that boundary. A more desirable behaviour is to only align voxels near the boundary, with more weight placed on the voxels the closer they are to the boundary. This can be achieved using a distance transform with a steep gradient at the boundary that quickly drops off away from the boundary. Therefore, a distance transform of the form ${y}=1-\tfrac{1}{1+{x}}$ was used. The distance transforms were masked out after a distance of 12 voxels from the boundary so that their effect in the registration is nullified once the distance from the structure boundary becomes too large to be relevant. Examples of the distance transforms generated are presented in figure 2.

#### 2.2.2. Vessels

The vesselness measure was used to capture and represent the vessel information. The vesselness measure (Frangi *et al* 1998) is a vessel detection approach that uses the Hessian matrix of each voxel to describe the curvature at that voxel. The eigenvalues of the Hessian can indicate if the voxel is part of a blob-like, tube-like, or plate-like structure, and the relative brightness or darkness of the structure compared to the background. Computing these values at multiple scales enables a vesselness value to be calculated for each voxel, which effectively measures how likely it is that the voxel belongs to a vessel. The PTK (Doel 2012) was used to calculate the vesselness.

Once generated, the two distance transforms and the vesselness image were concatenated to form a multi-channel (4D) image, with the lung boundary distance transform in the first channel, the airway boundary distance transform in the second channel, and the vesselness in the third. Examples of the vesselness images generated can be found in figure 2.

### 2.3. Registrations

The CT images were co-registered using the open-source NiftyReg registration software (https://github.com/KCL-BMEIS/niftyreg) which implements a B-spline based free form deformation (FFD) algorithm (Modat *et al* 2010). Good initial alignment of the source and target images is vital for the success of a deformable registration. The images were initially rigidly co-registered using the block-matching algorithm (Ourselin *et al* 2001) available in NiftyReg, using a strategy for bone anatomy alignment described in Veiga *et al* (2018). Diffeomorphic deformable registrations were then performed using the stationary velocity field parameterisation available in NiftyReg (Modat *et al* 2012).

In NiftyReg, transformations are parameterised by a control point grid. The FFD algorithm locally deforms the underlying control point grid of the image and interpolates the deformation field using cubic B-splines. The transformation is optimised using a conjugate gradient ascent optimiser. Diffeomorphic registrations have been implemented in NiftyReg using a log-Euclidean FFD approach where a spline model of a stationary velocity field is exponentiated to yield a diffeomorphism. This implementation results in invertible transformations that preserve the topology of the input images. These diffeomorphic transformations are performed symmetrically, with the backwards and forwards transformations being calculated simultaneously.

NiftyReg can perform multi-channel registrations. With multi-channel registrations, each input image (source and target) can consist of multiple channels, each holding complementary information. The similarity between the images is calculated independently for each channel and then summed. Different similarity measures can be used for each channel, and the channel similarities can be weighted relative to each other to control the influence that each channel has on the registration.

Optimisation of the registration parameters was carried out for those parameters that were expected to have the greatest effect on the registration results, namely: choice of image similarity measure and weight for each channel, choice of and weight of the penalty term, and spacing of the B-spline control point grid. Over 50 combinations of parameter values were examined and since each registration required multiple hours to complete, the optimisation was only performed on four representative test cases instead of on the full data set. Similarity measures considered were the SSD (sum of square difference), LNCC (local normalised cross correlation) and NMI (normalised mutual information), with relative weights for each channel ranging from 0.5 to 100. Bending energy and linear energy were considered for the penalty term, but ultimately the linear energy was not used as it penalises stretching and shearing, which are useful deformations for these registrations. The control point grid spacing tested ranged from 1 mm to 7 mm. The best parameters were chosen after evaluating the registration results visually and quantitatively by landmark registration error (see section 2.4 for how this was calculated). The results with lowest mean and median landmark error were selected to be closely visually inspected, and the parameters providing the best alignment in the structures of interest while also keeping to plausible deformations were favoured. Overall, it was observed that the registrations were robust to small changes in individual parameter values, therefore the most important aspect of the optimisation was achieving appropriate balance between the terms. It was also observed that changes in the control point grid spacing had a stronger regularising effect than changes in the regularisation term itself. Finally, the best results were given when the vesselness channel was weighed considerably higher than the lungs and airways, due to the large-scale structures being easier to align than the smaller and more detailed vessels. The best parameters were found to be the SSD with a weight of 1 for the lung and airways distance transform channels and the NMI with a weight of 2.5 for the vesselness channel; bending energy with a weight of 0.001 for the penalty term; an isotropic control point spacing of 5 mm.

The optimised parameters were then used to perform pairwise registrations for each of the 15 patient datasets, registering the baseline scan with the 12 month follow up. For 4 out of the 15 patients (one from each category), pairwise registrations were also performed between the baseline and remaining follow up scans (at 3, 6, and 24 months), in order to investigate the effect of temporal distance on registration accuracy.

### 2.4. Landmark placement and landmark registration error

**Figure 3.**
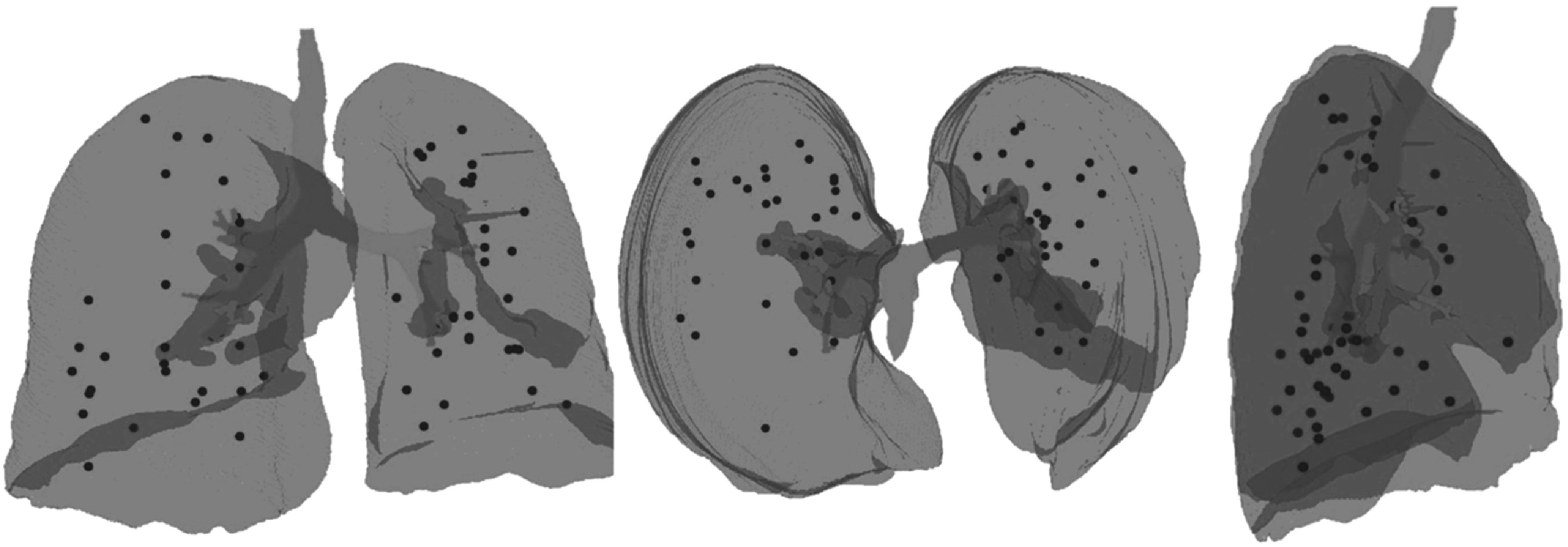
Coronal, axial and sagittal views of a 3D rendering of the baseline lungs and main airways of patient dataset with the landmark points in red.

In order to evaluate the registrations corresponding landmarks were manually identified in the images using the NiftyView application (https://github.com/NifTK/NifTK). Landmarks were placed on vessel and airway bifurcations. The landmarks were distributed over both lungs and for all datasets at least half the points were located in the ipsilateral lung, where larger changes were expected. Where possible, landmark points were concentrated close to the tumour (in the baseline) and RILD (in the follow up), as those are the areas of interest that must be assessed. However, in cases where the geometrical changes were substantial and the damage was particularly severe, it was impossible to identify matching landmark pairs in those areas of interest. As manual landmark identification is a time consuming and laborious task, landmarks were identified for all 5 timepoints in only 4 out of the 15 patients (one from each category). For the remaining 11 patients, landmark pairs were identified between the baseline and 12 month follow up scan only. An average (range) of 44 (38–65) landmarks were identified per CT. An example of landmark distribution for one dataset is available in figure 3.

Quantitative evaluation of the registrations is achieved through calculation of the post-registration landmark distance (*D*_*reg*_). *D*_*reg*_ is calculated as the Euclidean distance between the landmark points in the source image and the corresponding landmark points from the target image transformed by the registration result. Since the registrations were symmetrically performed in two directions, *D*_*reg*_ was calculated for both registration directions and averaged. For comparison, the pre-registration landmark distance (*D*_*pre*_) was also calculated as the Euclidean distance between the corresponding landmarks in the images without any deformable transformation being applied.

### 2.5. Intensity only registrations

To demonstrate the differences between the feature-based approach presented in this work and traditional intensity-based approaches, an intensity-based registration was performed for each of the 15 datasets using the 12 month follow up scan. The same diffeomorphic NiftyReg registration algorithm was employed but without the multichannel implementation. The original CT images were used as the inputs and parameters were kept consistent with the parameters of the multichannel registrations where appropriate (e.g. control point spacing, similarity measure (NMI), regularisation).

## 3. Results

### 3.1. Multichannel registrations

#### 3.1.1. 12 months

A summary of the numerical results of co-registering pre and 12 months post-RT scans using the proposed multichannel method is reported in table 1. Across all patients, the mean (standard deviation) *D*_*pre*_ is 15.95 (8.09) mm, and after deformable registration, *D*_*reg*_ decreases to 4.56 (5.70) mm. A clear improvement in the mean and median *D*_*reg*_ is noticeable for all 15 patients. For four patients, the mean *D*_*reg*_ is smaller than the corresponding mean slice thickness and for nine patients, their mean *D*_*reg*_ is less than twice their mean slice thickness. The median *D*_*reg*_ is less than the slice thickness for 12 out of 15 patients.

**Table 1.** Summary statistics for pre- and post-registration landmark distance.

Category	Patient No	No of landmarks	Mean slice thickness (mm)	Mean *D_pre_* (mm)	Mean *D_reg_* (mm)	Median *D_pre_* (mm)	Median *D_reg_* (mm)	St.Dev. (mm)
1	1	65	1.25	3.68	1.03	3.35	0.84	0.65
	2	50	2.50	14.47	2.77	10.29	1.97	3.41
	3	48	2.75	10.19	1.82	10.26	1.49	1.25
	4	42	1.00	7.50	1.13	4.78	0.68	2.64
2	5	48	3.75	16.97	3.13	13.78	2.05	3.69
	6	42	0.85	14.99	2.80	14.46	0.79	5.46
	7	43	1.25	24.59	7.69	25.23	4.77	8.57
	8	41	2.50	15.54	2.18	16.53	1.39	2.76
3	9	36	5.00	25.57	8.38	22.48	2.44	9.89
	10	38	2.00	20.36	5.97	16.83	1.86	8.08
	11	43	5.00	12.01	6.54	6.27	1.90	10.25
4	12	38	2.10	13.81	6.16	12.23	5.19	4.47
	13	38	1.25	20.45	6.21	17.47	1.47	8.58
	14	45	5.00	16.65	6.34	13.93	1.91	7.81
	15	39	3.00	22.46	6.27	22.46	2.61	8.00

**Figure 4.**
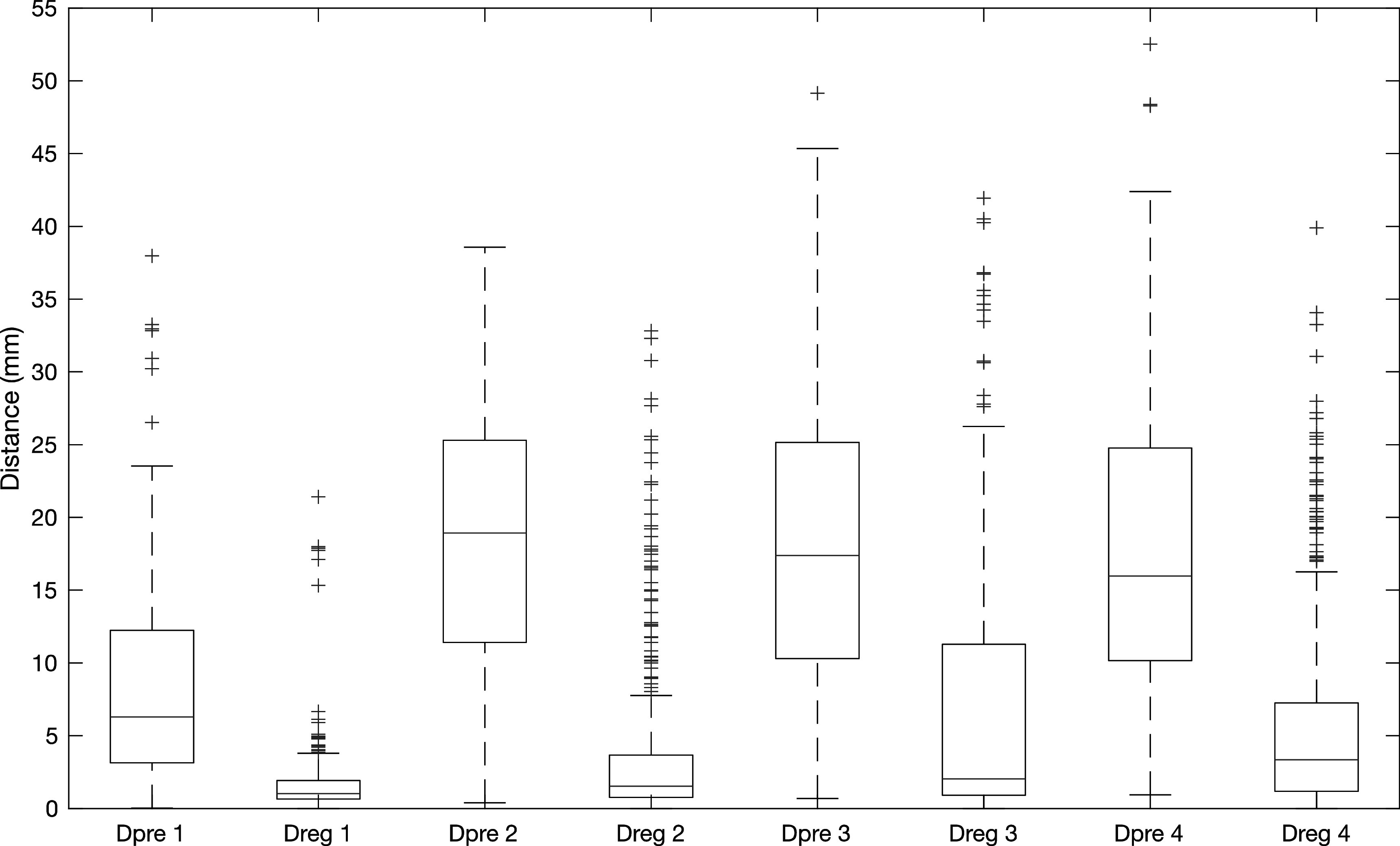
The distribution of *D*_*pre*_ and *D*_*reg*_ for each data category presented in box plots. The red line represents the median and the bottom and top edges of each box plot represent the 25th and 75th percentiles respectively. The whiskers extend to the most extreme data points that are part of the distribution and the outliers are points beyond ±2.7 std and are plotted with the + symbol.

**Figure 5.**
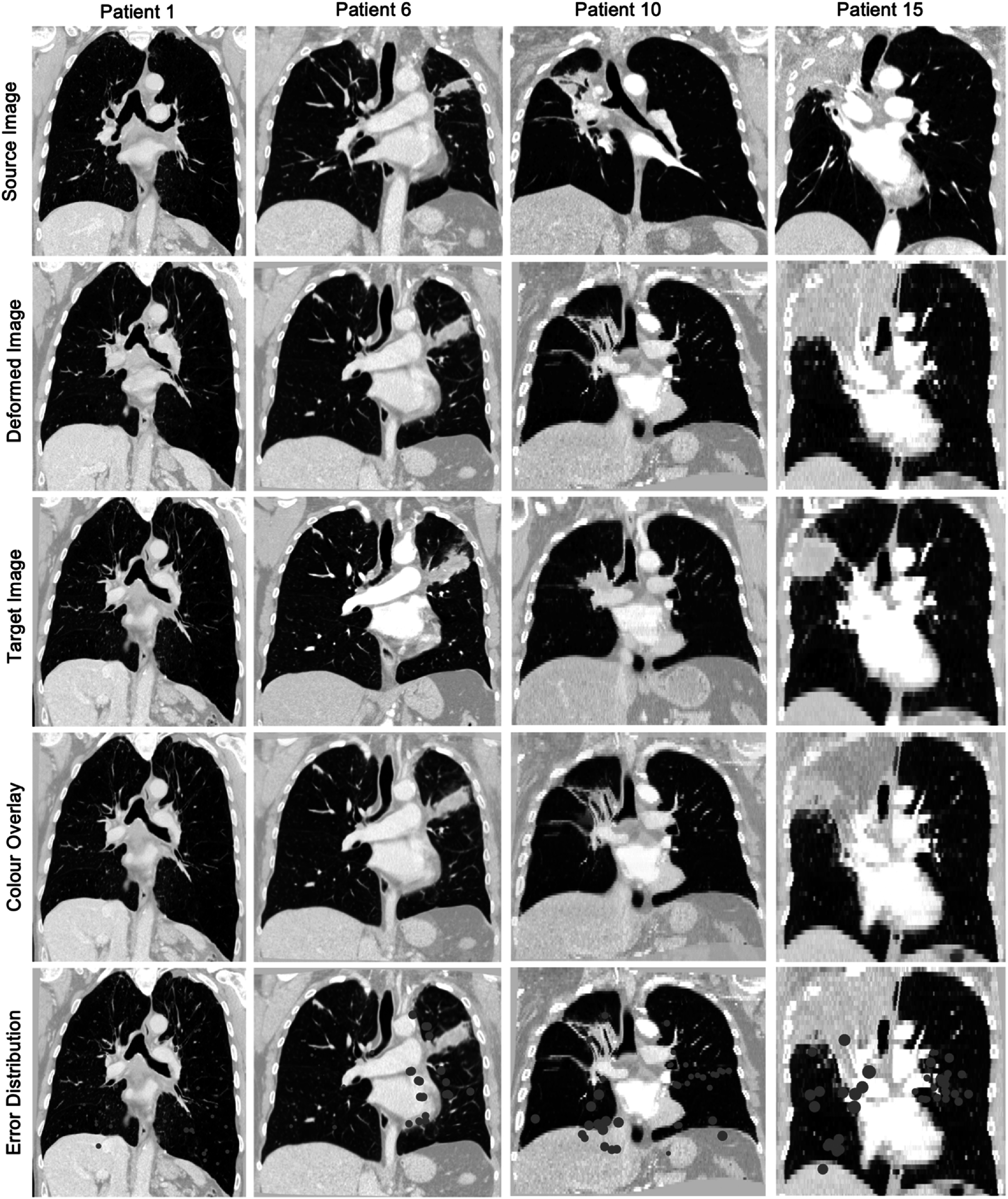
From left to right: example CTs of patients of increasing difficulty. Top to bottom: coronal slices from source CT (12 month follow up), target CT (baseline), deformed CT, colour overlay of target (red) and deformed CT (cyan), landmark points displayed over the deformed CT where the size of the landmark point corresponds to the size of *D*_*reg*_ at that point.

In figure 4, the distributions of landmark errors before and after the registration are presented for all four categories. In all cases, a decrease in the median, 25th percentile and 75th percentile distance as well as a narrowing of the distributions can be observed. As expected, in the two easier categories (1 and 2) corresponding to cases with mild or moderate changes, higher registration accuracy was achieved compared to the categories that correspond to cases with more extensive damage (3 and 4), as demonstrated by the lower *D*_*reg*_. The relative improvements between the *D*_*pre*_ and *D*_*reg*_ are also larger for the lower categories, as can be seen in table 1. It is important to acknowledge the presence of outlier *D*_*reg*_ values in these results, with a number of individual *D*_*reg*_ over 25 mm for categories 2–4, as can be seen in figure 4. These errors are mainly present in the more difficult cases and usually in the ipsilateral lung where damage is present and the deformations to recover are the largest. Figure 5 shows examples of results from each category.

When looking at the *D*_*pre*_ and *D*_*reg*_ for each patient, it becomes apparent that even though some patients have a similar mean *D*_*pre*_, the *D*_*reg*_ can vary substantially. For example, patient 2 has a mean *D*_*pre*_ of 14.47 mm and a mean *D*_*reg*_ of 2.77 mm, but patient 12 has a lower mean *D*_*pre*_ of 13.81 mm and a mean *D*_*reg*_ of 6.16 mm, which is more than double that for patient 2. This suggests that *D*_*pre*_ may not be the only factor that determines the success of a registration. Other important factors include the type of damage, its location, and how extensive it is. Some cases might have very large *D*_*pre*_ but that may be due to global rotations or shearing of the lung tissue. These global changes are easier to align compared to more localised and less well-defined changes such as the appearance of consolidation or atelectasis.

#### 3.1.2. Qualitative observations

In addition to the quantitative evaluation performed above, it is vital to visually inspect the registered images in order to gain insight into where and why the registrations failed to align the two scans. Qualitative observations generally agree well with the quantitative results. As expected, the contralateral lung is very well aligned for almost all patient datasets. The largest errors are always located in the ipsilateral lung and usually close to areas of damage, as can be seen in the examples in figure 5. The lung boundaries are well aligned for all patients, both in the ipsilateral and contralateral lungs, which can be seen in the colour overlays in figure 5. This is especially important in the case of patient 15, where even though the upper right lobe has completely collapsed, the lung boundary is correctly aligned. The main airways are also generally well aligned. The lung boundaries and airways are aligned well even in cases with extreme deformation between scans, such as patient 10 and 15 shown in figure 5. While the achieved registration results are promising, there are some regions where the alignment could still be improved, especially in cases with extreme deformations. Local misalignment may remain, for example, at smaller airways and vessels, as can be seen in the ipsilateral lungs of patients 10 and 15 (see figure 5).

It is important to highlight that the aim of the registrations is not always to make the deformed image look exactly like the target image. For our application, the goal is to align corresponding regions of the images such that the local changes that have occurred between them can then be studied. This is most prominent in examples like patients 10 and 15 in figure 5, where the scans appear reasonably well aligned and the differences between the deformed scan and target image represent non-deformable changes characteristic of RILD (atelectasis, consolidation) rather than registration errors.

#### 3.1.3. Serial time-point analysis

**Figure 6.**
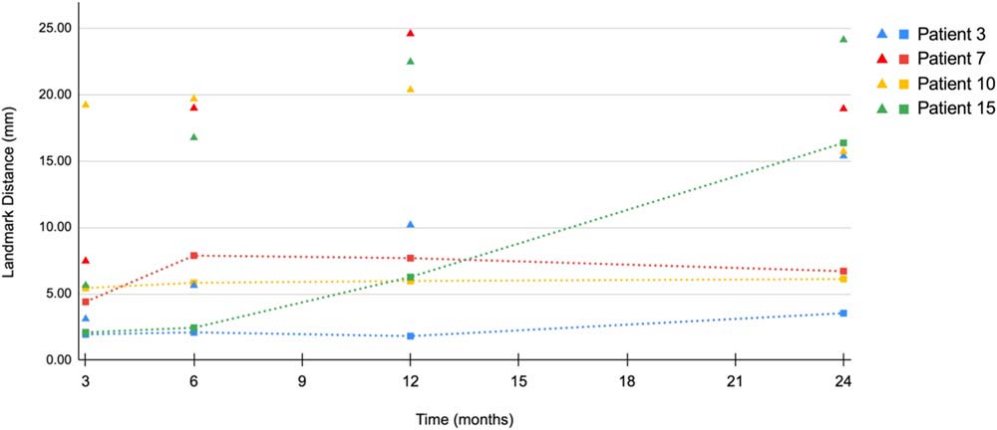
Mean *D*_*pre*_ (triangles) and *D*_*reg*_ (squares) for patients 3, 7, 10 and 15 (increasing difficulty) at 3, 6, 12, 24 months post-RT.

**Figure 7.**
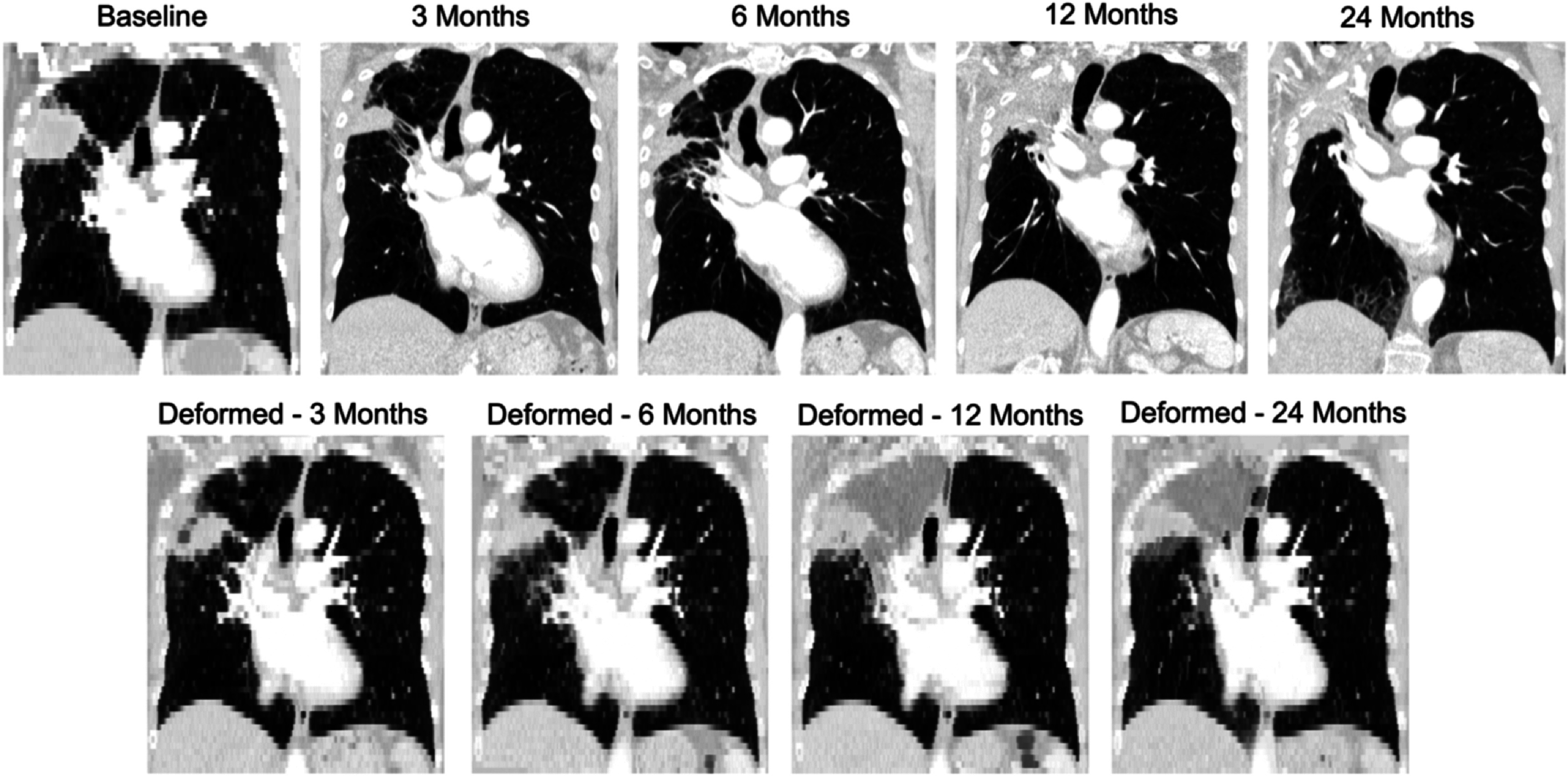
Top row: patient 15 at five time points. Upper right lobe collapse first appears in 12 month scan and remains in the 24 month scan. At 24 months new texture appears in the lower right lobe. Bottom row: colour overlays between baseline CT and registration results from registering all time points.

Temporal evolution of the mean *D*_*reg*_ for the four cases of increasing difficulty included in this analysis is presented in figure 6. For three out of the four cases, the landmark error *D*_*reg*_ is relatively stable with a general trend to slightly increase over time. This is an expected trend, as the effects of RILD tend to become more pronounced when chronic changes settle in. For patient 15 there is a considerably abrupt increase in *D*_*reg*_ at 24 months. The increased error is likely due to the extreme anatomical change present in the 24 month scan, even more so than the 12 month as can be seen in figure 7. These extreme changes also affect the accuracy of the landmark point selection. This higher *D*_*reg*_, therefore, could partially reflect user errors in landmark selection rather than significantly poorer registration accuracy. The lowest errors across all patients are at 3 months, with all of them below 5.5 mm and their mean at 3.5 mm. Even though this is the time point where radiation pneumonitis appears, its effects seem to have a less negative effect on the registrations compared to the chronic effects of RILD. This is substantiated by Veiga *et al* (2020) where it was observed that in the long term, parenchymal change is accompanied by anatomical distortion but at 3 months, less distortion is present.

### 3.2. Multichannel versus intensity-based

**Figure 8.**
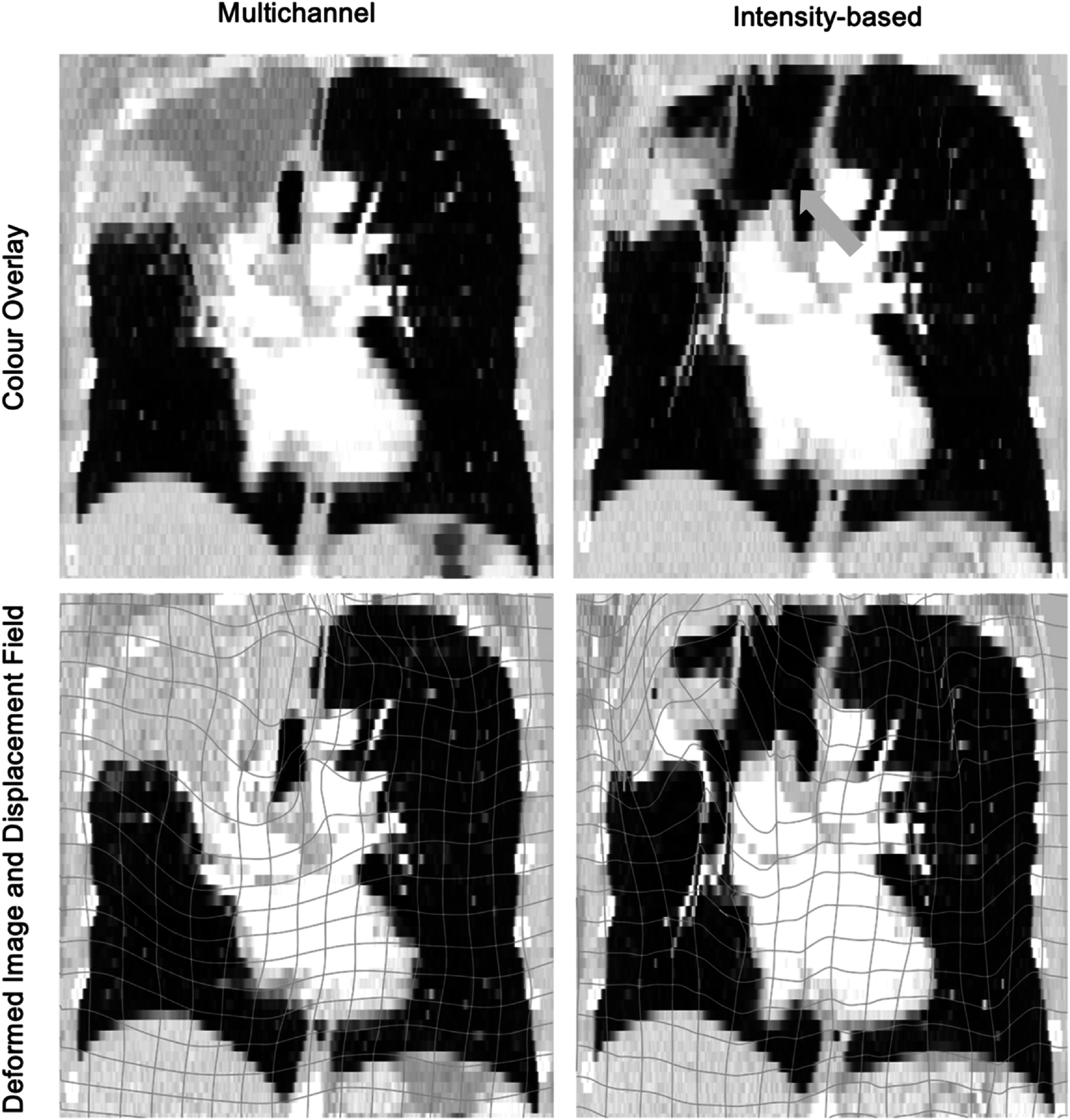
Multichannel versus traditional intensity-based registration results for patient 15. The anatomically implausible deformations of the intensity approach are apparent in the bottom right image, where the displacement field of the registration is displayed over the registration result. The trachea in the deformed image has been stretched to the size of the upper right lobe in the baseline scan. The green arrow in the colour overlay (top right) points to the airway boundary of the baseline scan that has been stretched and is therefore missing from the deformed image. In comparison, the displacement field in the multichannel result is smooth, with no implausible or extreme deformations present.

The overall mean (standard deviation) *D*_*reg*_ for the intensity registrations is 7.90 (8.97) mm, which is considerably higher than for the proposed multichannel registrations at 4.56 (5.70) mm. For some cases in categories 1 and 2, where the deformations are smaller and there are no extreme anatomical changes (atelectasis, cavitation, etc), the intensity approach performs similarly to the proposed multichannel approach, aligning the baseline and 12 month scans well, with *D*_*reg*_ close to that of the multichannel registrations. However, that is not the case for the remaining cases with more complex deformations and anatomical changes. The shortcomings of the intensity approach can mainly be seen in the cases where lung tissue or structures expand, contract or change beyond visual recognition, for example where there is collapse or extensive consolidation. An example of this can be seen in the registration result of patient 15 (figure 8) where in the registration result, the airway in the follow up scan has been extremely stretched to align with the upper right lobe and tumour boundaries of the baseline scan. These deformations are not only anatomically implausible but also fail to align corresponding regions of the lungs.

## 4. Discussion

Baseline and 12 month post-RT CT pairs from 15 patient datasets with RILD were split into four categories according to expected registration difficulty and registered using the software NiftyReg. Diffeomorphic feature-based registrations were performed where only salient features that were consistent between the two images of a pair were used. These features included the lung boundaries, main airways and vessels. The CT intensity information was disregarded in order to minimise the effect of severe, non-deformable tissue damage on the registration results. The results were evaluated quantitatively by calculation of the post-registration landmark distance at a number of manually identified anatomical landmark pairs distributed across both lungs. Quantitative findings were critically evaluated using visual inspection. We have demonstrated that our proposed method is suitable for aligning pre-RT and follow-up CTs from lung cancer RT patients exhibiting considerable anatomical changes due to RILD. This will facilitate future investigations into longitudinal changes to the local parenchymal tissue occurring due to RILD, and to relate these to the RT dose that was delivered.

Improvement in image alignment was achieved for all patient datasets. Categories 1 and 2 cases saw the biggest improvements in alignment and the best overall results. Categories 3 and 4 cases saw smaller quantitative improvements in comparison, however visually notable improvements in alignment were observed for all patients. Overall, good local alignment was achieved for all patients. The performance of the registrations for other timepoints (3, 6, 24 month follow-up) was investigated for four patient datasets. It was found that the landmark distances *D*_*reg*_ mostly increased over time, as is expected due to more the extreme deformations of chronic RILD. The lowest registration errors for all patients were measured when registering 3 month scans.

Results from the proposed multi-channel feature-based methodology were compared to results from a traditional intensity-based DIR methodology. Our results indicate that for co-registration of serial CT scans in the presence of RILD our novel method is superior to the traditional intensity-based approach. The intensity-based registration achieved comparable results to our method for patients with modest anatomical changes between baseline and 12 months but produced highly implausible results when the damage was more pronounced.

Even though the intensity information of the CT is excluded from the registrations in order to minimise the effects of the extensive tissue damage on the results, large geometric changes also occur in the vasculature of the patients. For example, in atelectatic parts of the lung, vessels collapse under the pressure of the surrounding tissue, making them unrecognisable in a CT. If a region of the lung is damaged by radiation and is caused to shrink, consolidate or move, vessels supplying that region of the lung will follow along. This means that there is not a perfect correspondence even between ‘consistent’ anatomical features when so much time has passed after radiotherapy. These geometric changes also affect the main airways and lung boundaries but, in those cases, correspondence is considerably easier to establish due to the larger scale and less repetitive nature of these structures compared to vessels. This is why the lung boundaries and main airways are remarkably well aligned even in the most difficult cases, but small-scale vessels and deeper airways can be misaligned. This also has a considerable impact on our ability to accurately evaluate the accuracy of the registrations. Landmark pair identification is already a challenging and laborious task but even more so in datasets that carry such extreme anatomical changes between time points. In many cases, it is impossible to identify corresponding landmarks between scans in some regions, which means that it is also impossible to quantitatively assess how successful the registration is in those regions. Therefore, visual inspection of the registration results thus becomes very important in challenging applications such as ours. Even so, it is not a perfect way to completely capture all facets of the results.

Comparing our results with those achieved in other studies is informative but can also be misleading. In the literature, even when the word ‘longitudinal’ is used to describe the temporal distance between scans, this usually means 3 months post-RT at most and even in these cases, patients with dramatic geometric changes between time points, and especially atelectasis, are usually excluded. In the most comparable piece of work by Guy *et al* (2018), some level of atelectasis was present in all the scans. In that study a mean (st. dev.) of 2.60 (0.90) mm landmark error was achieved, however, the scans were only a few weeks apart, as mid-treatment scans were used. This means that geometric changes that come with chronic RILD have not yet developed, making the registration process less complex. Additionally, their mean landmark distance before DIR was 9.93 mm whereas for our data it was considerably higher at 15.95 mm. In Cunliffe *et al* (2015), mean errors of 2.5 mm and 4.6 mm were found when registering pre-RT and 3 month post-RT CTs in the presence of damage by two different DIR algorithms, namely Demons and Morphons. However, the analysis was carried out only in a number of small regions across the lungs, and the PTV, a region that is hard to align and can give high errors, was excluded. On the other hand, we tried to focus on landmarks around the tumour and areas of RILD, as, although these are the regions that are hardest to align, they are also the ones of most interest. In Spijkerman *et al* (2014), the best performing, medium, and worst performing groups had errors of 3.8 mm, 4.5 mm and 8.0 mm respectively. Once again, the temporal distance between scans was only about 4 months. The EMPIRE10 challenge was a chest CT registration challenge held at MICCAI 2010 (Murphy *et al* 2011). Some teams managed to obtain low landmark errors of <1 mm, however, only 8 out of 30 scan pairs available were longitudinal (9–14 months between baseline and follow-up scan) and no radiation damage was present, meaning that standard intensity-based approaches could be successfully applied.

The mean landmark error for our 3 month registrations of 3.5 mm might be a more appropriate comparison with the results in the literature, however, this number is only based on four datasets so further evaluation is needed. When taking into consideration that the scans used in our study include later follow-up time-point scans, display more pronounced anatomical and geometrical changes due to RILD, and that our evaluation deliberately tried to include more landmarks from the most challenging regions, we consider our results competitive to those presented in the literature. Furthermore, we expect that our approach would achieve better results than other published methods on scans from 6 months or later follow-up, that are of most interest when studying chronic effects of RILD.

This study was limited by the need for detailed manual editing of the lung segmentations as accurate automatic segmentations of this data were not available at the time of this study. Currently, we are developing an automatic lung segmentation that can successfully be applied to RILD datasets and we aim to utilise it for future studies once it has been validated. Lobe segmentations would have also been included if available as they would provide additional corresponding elements throughout the lungs. However, current lobe segmentation algorithms do not yet perform well on scans with severe damage, especially atelectasis, or on scans with large slice thicknesses such as some of those included in this study. An attempt at automatically generating them using the pulmonary lobe segmentation algorithm published by Xie *et al* (2020) was unsuccessful when atelectasis was present in the scans.

In the future we intend to develop a fully automated pipeline for registrations. This will require methods to accurately and robustly segment the salient features without any manual intervention, possibly including additional structures such as lobe fissures and deeper airways. These automatic segmentation methods will need to be thoroughly validated, and the sensitivity of the registration results to the segmentation accuracy will be investigated and compared to the results presented in this paper. We will also explore the use of weakly supervised deep learning based registration approaches (Fu *et al* 2020), that can take advantage of features and structures that have been delineated in the training data, but are not required when applying the trained network to new, unseen data.

## 5. Conclusion

In this work, we proposed a novel registration methodology tailored to co-register longitudinal CT scans in the presence of RILD. We have demonstrated that our method is able to successfully align 12 month follow up scans with pre-radiotherapy scans, even in the presence of large anatomical changes such as consolidation and atelectasis. The method was also shown to perform well on follow-up scans acquired at 3, 6, and 24 months after radiotherapy. The quality of the registration varies with the extent of anatomical and geometrical changes characteristic of RILD present in the scans. This work will facilitate future work studies aiming at better understanding the local parenchymal tissue changes caused by RILD, as well as provide insights into dose-response relationships.
